# The development and progress of health literacy in China

**DOI:** 10.3389/fpubh.2022.1034907

**Published:** 2022-11-07

**Authors:** Yuanyuan Li, Xiaofeng Lv, Jun Liang, Hengjin Dong, Changgui Chen

**Affiliations:** ^1^Science and Education Department, Hangzhou Ninth People's Hospital, Hangzhou, China; ^2^Center for Health Policy Studies, School of Public Health, Zhejiang University School of Medicine, Hangzhou, China; ^3^IT Center, Second Affiliated Hospital, School of Medicine, Zhejiang University, Hangzhou, China; ^4^College of Medical Technology and Information Engineering, Zhejiang Chinese Medical University, Hangzhou, China; ^5^Key Laboratory of Cancer Prevention and Intervention, National Ministry of Education, School of Medicine, Zhejiang University, Hangzhou, China; ^6^General Practice, Hangzhou Ninth People's Hospital, Hangzhou, China

**Keywords:** health literacy, health promotion, health education, health progress, China

## Abstract

Limited health literacy is a serious public health problem. It is strongly associated with increased hospital admissions and readmission, poorer self-management, and health outcomes. It can lead to poor management of chronic disease, lower health care quality, increased mortality, and higher healthcare expenditures. Understanding China's current situation and the progress of health literacy levels are critical to achieving practical solutions for improving population health. This paper intended to provide a concise overview of the key milestones and specific practices in health literacy in China. We summarized the characteristics and changing profile of health literacy from 2008 to 2020 in China. We developed an intervention framework based on social ecosystem theory for improving health literacy in China. Meanwhile, some multi-level actionable recommendations were proposed. The study revealed that China has made progress in improving health literacy in the last decades. Health literacy levels increased from 6.48% of the population in 2008 to 23.15% in 2020. Geographic disparities were substantial. The East performed better health literacy than the Central and West, and cities had higher adequate health literacy than rural areas. Social development index, age, and education level were highly associated with health literacy. A global joint effort to improve health literacy will be required. And we advocate a whole-of-society approach that involves the participation of the entire ecosystem around the targeted population.

## Introduction

Health literacy was first introduced in the 1970s and had considerable attention worldwide in recent years in public health (1). The World Health Organization (WHO) defined it as the personal knowledge and competencies that enable people to access, understand, appraise and use information and services to promote and maintain good health for themselves and those around them (2). Health literacy is a comprehensive reflection of economic and social development and is influenced by various factors, including politics, economics, education, society, culture, and the health development level (3). The level of health literacy is a strong predictor of a person's health outcomes. Low health literacy has been associated with riskier health choices, risker behaviors, higher readmissions, and poorer health status. It can lead to poor management of chronic disease, increased morbidity, and premature death. Additionally, it can significantly drain the financial resources of the health system (4). Effective responses to health literacy issues can improve health outcomes and reduce health inequities. Improving the level of health literacy could produce potential economic savings of ~8% of total costs (5). WHO advocates that all countries should make an effort to promote health literacy to ensure the realization of the Millennium Development Goals. Policies and practices should identify health literacy issues and implement the targeted responses.

In China, we use the Chinese Health Literacy Scale to measure health literacy (6). It was developed by experts from public health, health education and promotion, and clinical medicine. And it was designed based on the “Chinese Resident Health Literacy—Basic Knowledge and Skills (Trial)” issued by the National Health Commission of the People's Republic of China in 2008. It contains three domains: basic knowledge and attitudes (BKA), healthy behavior and lifestyle (HBL), and health-related skills (HRS). The three domains covered 80 items and six aspects, including safety and first aid (SFA), scientific views of health (SVH), health information (HI), infectious diseases (ID), chronic diseases (CD), and primary medical care (PMC). The health literacy level refers to the proportion of people with basic health literacy in the total population. Criteria for determining basic health literacy: the health literacy measure questionnaire scored 80% or more of the total score. The health literacy level was 25.4% in 2021 in China, which means that an estimated 75% of adult Chinese (ages 16–69) lack the capacity to obtain, understand and act on health information and services, and also the ability to make appropriate health decisions on their own (7). Furthermore, China still faces daunting challenges in non-communicable diseases, which are driven by unhealthy dietary behaviors, environmental exposure risk factors, industrialization, urbanization, and a rapidly aging population (8). Taking a thorough understanding of the current situation and the characteristics of the health literacy level is critical for identifying priorities to organize them into a comprehensive framework for improving health literacy in China.

This study aimed to provide a concise overview of the promotion of health literacy in China from 2008 to 2020 in China, including the current situation, characteristics, changing profile, and key milestones and practices in improving health literacy. In addition, we proposed a social ecosystem-based intervention framework and offered some actionable recommendations for addressing the health literacy issue in China. We hope this study could offer some practical ideas for disseminating health inequities, improving population health, and contributing to making universal health coverage and Millennium Development Goals achievable.

## Methods

To understand the progress of health literacy in China, we conducted a scoping review. We collected and analyzed the policy files and data relevant to health literacy. We searched for these files and data on government departments' websites, including the State Council of the People's Republic of China, the National Health Commission of the People's Republic of China, the National Bureau of Statistics of the People's Republic of China, and the provincial Health Commission of China. In addition, to better develop the intervention framework, we searched Pubmed, China Academic Journals full-text database (CNKI), and Wangfang Database, and reviewed articles relevant to the broad scope of this review. “Health literacy,” “eHealth literacy,” “internet literacy,” “health intervention,” “health education,” “health promotion,” “health progress,” and “China” were used as search terms. A narrative synthesis summarized the results of included files and data. Additionally, we conducted spearman's rank correlation analysis to examine the association between the regional health literacy level and the social development index (SDI).

## The development and progress of health literacy in China

Since the concept of health literacy was introduced in 2005 in China, there has been a comprehensive effort to enhance the study and promotion of health literacy. We reviewed the policy documents and health literacy reports issued by the national and provincial Health Commission. Based on this, in this section, we summarized the key milestones in the promotion of health literacy in China, the characteristics, and the changing profile of the Health Literacy level in China from 2008 to 2020.

### Key milestones in the promotion of health literacy in China

Table 1 summarized the key information on the development of health literacy issued by the Chinese government. In 2008, China launched the nationwide health literacy promotion action, and released the first government document “Chinese Resident Health Literacy—Basic Knowledge and Skills (Trial).” It covered 66 aspects of health literacy including basic knowledge and beliefs, healthy lifestyles and behaviors, and basic skills (9). Based on this document, we issued the “Chinese Resident Health Literacy Promotion National Action Plan (2008–2010),” developed the health literacy scale, and conducted the first national survey of health literacy (10). Subsequently, the State Council of the People's Republic of China released the “National Basic Public Health Services (NBPHS).” It provided 22 in nine categories of free basic public health services, and health education was one of the independent services as the key rule for improving citizens' health literacy (11, 12).

**Table 1:** Key milestones of the development of health literacy, in China.

**Year**	**Key milestones**
2008	Released “Chinese Resident Health Literacy Promotion National Action Plan (2008–2010)”, released Chinese Resident Health Literacy—Basic Knowledge and Skills (Trial), and initiated the first-ever national assessment of the health literacy of Chinese residents. Implemented the National Tobacco Control Mass Media Campaign in China.
2009	Released “National Basic Public Services of the Peoples Republic of China”, health education is one of the independent services.
2010	Conducted health literacy index system research.
2012	Issued “Twelfth Five-Year Plan for National System of Basic Public Services of the People's Republic of China”; Issued the “Twelfth Five-Year Plan for Health Sector Development”. Improving the health monitoring system, initiating a central subsidy program for health promotion action; carrying out comprehensive health education, conducted continuous health literacy monitoring.
2014	Released “National Health Literacy Promotion Action Plan (2014–2020)”, planning the rate of national health literacy is aimed to increase to 20% by 2020.
2016	Issued “Health China 2030”, planning the rate of national health literacy is aimed to increase to 30% by 2030.
2019	Released “Health China Programme (2019–2030)”, and initiated health China action.
2020	Issued “Chinese Resident Ecological Environment and Health Literacy” by the Ministry of Ecology and Environment, emphasize the importance of the ecological environment and its impact on health literacy.

In 2012, the State Council of the People's Republic of China initiated a central subsidy program for health promotion action, advocating establishing a gradual and stable health literacy monitoring system (13, 14). In 2014, China released the “National Health Literacy Promotion Action Plan (2014–2020).” And this was a programmatic document for the in-depth development of health promotion and health education. It clarified the goals and tasks of health literacy promotion in the future, intended to increase adequate health literacy to 20% by 2020 (15). In 2016, “Health China 2030” was launched, and intended to increase the national health literacy level to 30% by 2030 (16). Meanwhile, the ecological environment and its impact on health literacy became increasingly important, and the Ministry of Ecology and Environment released the “Chinese Resident Ecological Environment and Health Literacy” in 2018 (17).

### The characteristics and the changing profile of health literacy level in China

**Figure 1:**
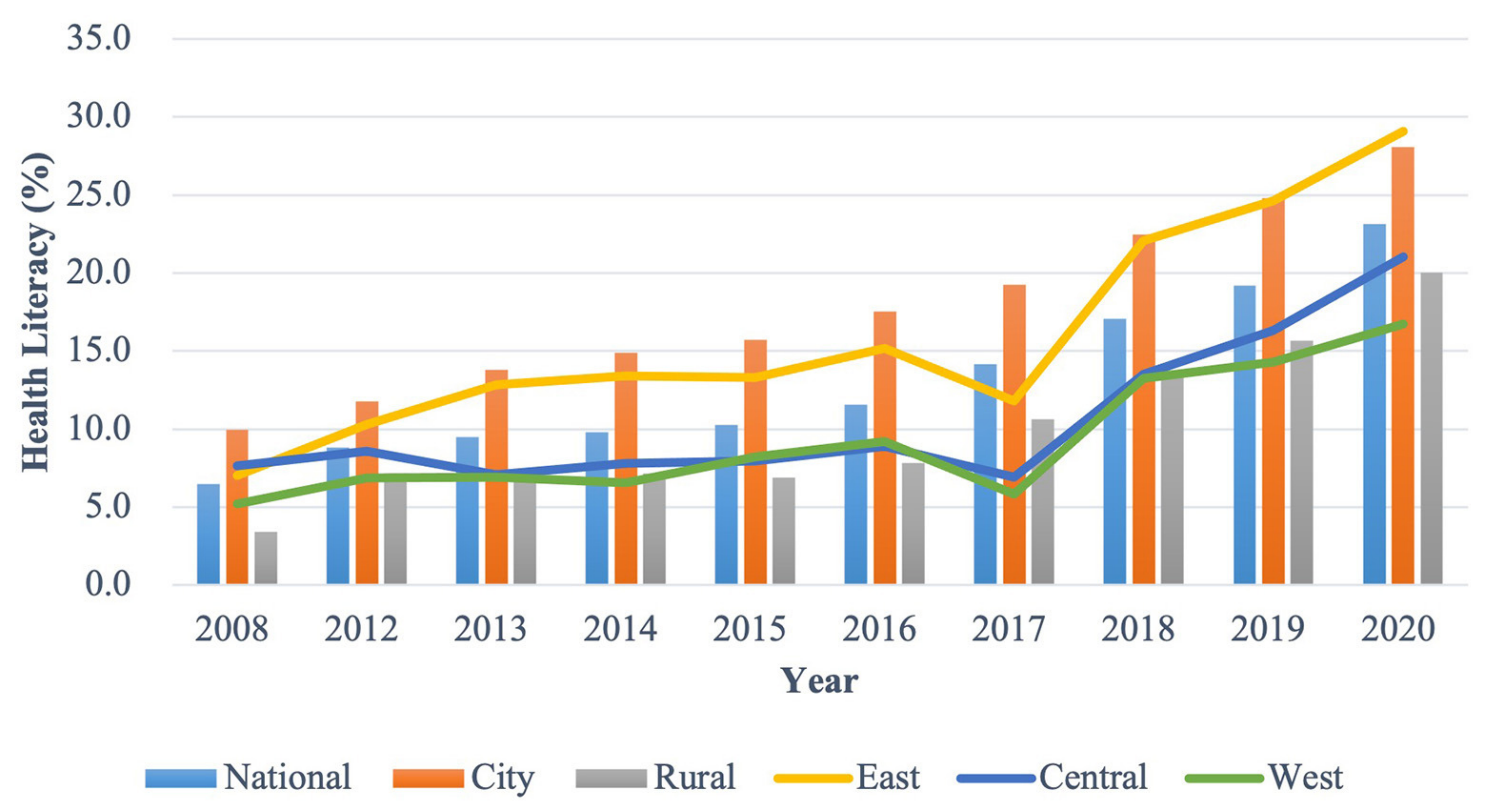
The distribution of the overall health literacy level by year and region, in China.

**Figure 2:**
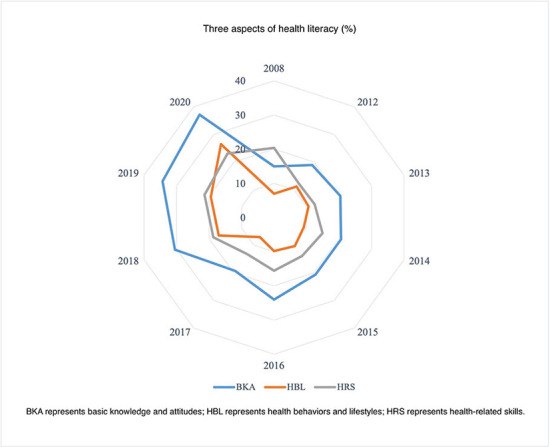
The distribution of the three aspects of health literacy, in China.

**Figure 3:**
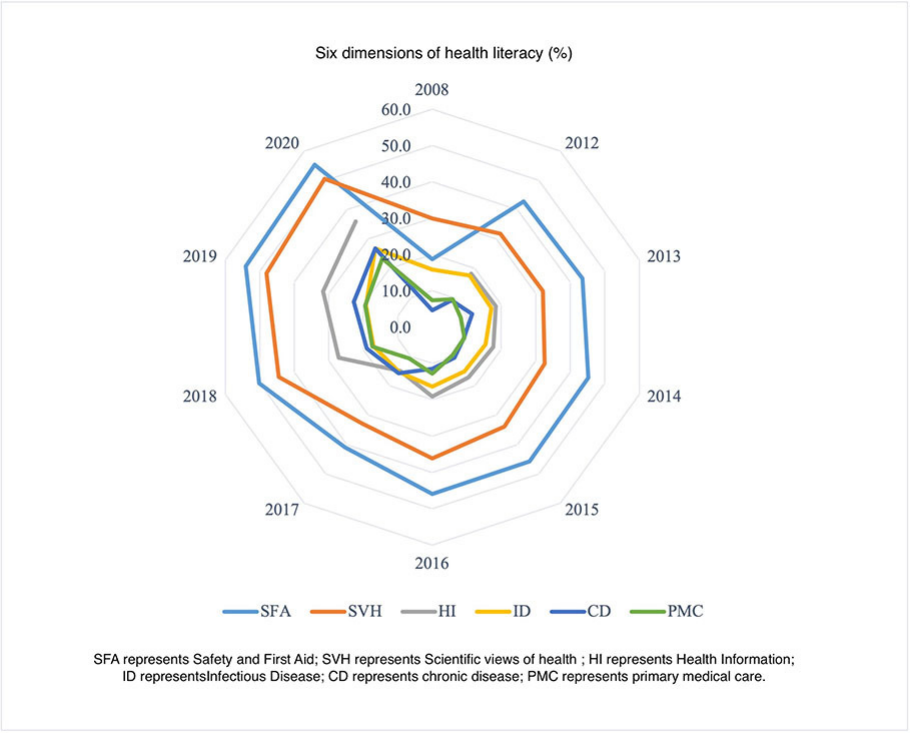
The distribution of the six dimensions of health literacy, in China.

**Figure 4:**
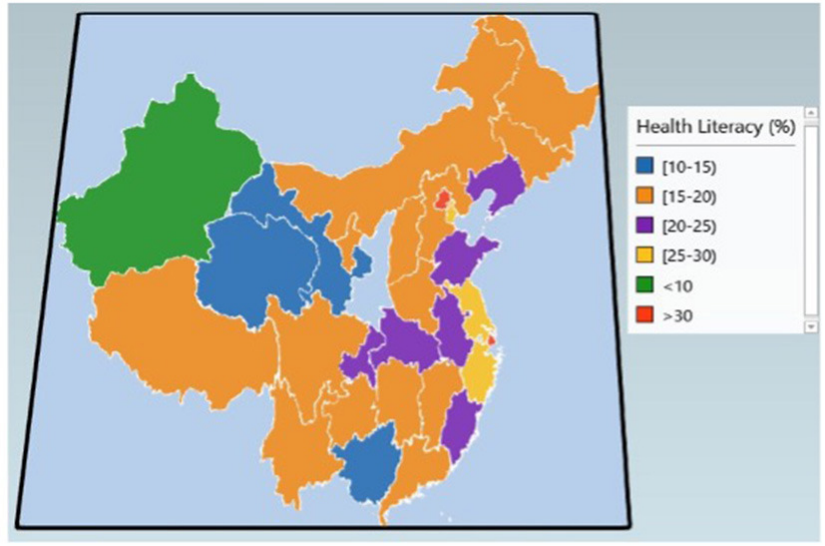
Geographical distribution of health literacy in 2019, in China.

We summarized the characteristics and changing profile of the health literacy level in China from 2008 to 2012 (Figures 1–3). Additionally, we presented the association between the regional health literacy level and the SDI (Figure 4).

Figure 1 showed the overall health literacy level from 2008 to 2020 by year and region. The national overall health literacy level increased from 6.48 to 23.15%, with a 1.13% of average annual growth rate. The city had a higher level than rural areas, with it increasing from 9.94 to 28.08% and from 3.43 to 20.02% in the urban and rural areas, respectively. East had higher health literacy levels (ranging from 7.67 to 21.01%) than the Central (ranging from 7.67 to 21.01%) and West areas (ranging from 5.23 to 16.72%).

Figures 2, 3 displayed the three aspects and six dimensions of health literacy level. Significantly, they all increased over time. Regarding the three aspects, the health literacy level of BKA was comparatively higher than HBL and HRS. From 2008 to 2020, the BKA literacy increased from 14.97 to 37.15%, while HBL and HRS literacy increased from 6.93–26.44 to 20.39–23.12%, respectively. Regarding the six dimensions, SFA literacy had the highest level (ranging from 18.70 to 55.23%), closely followed by SVH (ranging from 29.97 to 50.48%). PMC and CD literacy had the lowest level among the six dimensions, ranging from 7.43–23.44 to 4.66–26.73%, respectively. The HI and ID literacy increased from 18.16–35.93 to 15.86–26.77%, respectively.

Figure 4 addressed the geographical distribution of the overall health literacy level in 2019 in China. We used the spearman rank correlation analysis to examine the association between health literacy and SDI. It presented a statistical significance that health literacy was highly associated with SDI (Supplementary Figure 1). The Spearman's correlation coefficient was 0.76. Areas with higher SDI have higher levels of health literacy. Beijing and Shanghai had the highest health literacy level (34.30 and 32.31%), followed by Zhejiang (29.49%), Tianjin (26.29%), and Jiangsu (25.33%). Xinjiang had the lowest health literacy level (9.72%).

## Key practices in the promotion of health literacy in China

In the last decades, China adopted comprehensive intervention to improve health literacy. In this section, we reviewed policy documents, China Health Statistics Yearbook (2012–2020), and some published papers, and presented the key practices in the promotion of health literacy, including developing national basic public health services, health education and promotion, tobacco control, continuous health literacy monitoring, and holding special revenue fund.

### National Basic Public Health Services

The Chinese State Council has released comprehensive actions to improve health literacy. The NBPHS was first issued in 2009. It was an essential public health service provided freely by the Chinese Government to address the main health problems of all the Chinese residents, especially focusing on children, pregnant women, the elderly, and patients with chronic diseases (11). In 2022, its coverage categories increased from 22 public services in nine categories in 2009 to 54 services in 14 categories, defining health education and health literacy promotion action as the two independent services. Health education provides five public health services, including providing health education materials, setting up a health education bulletin board, carrying out public health consultations, holding health knowledge lectures, and providing individualized health education. Health literacy promotion action provides six public services, including building a health promotion county, carrying out science popularization, building a health promotion hospital and smoking cessation clinic, monitoring the health literacy and tobacco epidemic, setting up a health hotline (12,320), and promoting health education for key disease, fields, and key populations.

### Health education and promotion services

The key initiative for improving citizens' health literacy is health education. China established professional health organizations (PHO). These organizations provided comprehensive health education and promotion services. Table 2 addressed the detailed national health education and promotion services that were collected from China Health Statistics Yearbook (18). From 2012 to 2020, PHO provided a large number and useful health technical and policy advice and public health education activities. They worked with the media together to build digital health communication and health science popularization platform. They disseminated the basic knowledge and skills of citizens' health literacy through a large number of brochures, short messages, and health promotion supplies. Meanwhile, they established a team of health science experts to organize a series of health China activities and opened national hotlines to provide professional advice for health promotion.

**Table 2:** National services of health education and promotion by the professional health organizations from 2012 to 2020 in China.

**Year**	**Health education services**				**Production of health promotion materials**				**Web hosting (columns)**	**Total health education trainees (million visits)**
	**Technical and policy advice (times)**	**Media cooperative programs (times)**	**Public health education activities (times)**	**Media cooperative broadcasting health promotion information (times)**	**Brochures (million copies)**	**Video products (million copies)**	**Short message service (million copies)**	**Health promotion supplies (million copies)**		
2012	57,955	206,959	77,599	666,283	373.9	2.4	210.3	25.5	787	1.1
2013	6,856	4,759	47,245	183,539	367.0	1.0	144.5	33.7	884	1.2
2014	9,305	6,827	74,034	407,400	396.4	0.9	155.0	33.0	987	1.3
2015	10,700	5,589	79,283	356,369	395.7	1.3	121.1	42.8	1,050	1.3
2016	10,461	4,764	66,780	256,107	421.4	2.0	141.0	47.9	1,198	1.6
2017	10,439	4,176	61,507	213,469	380.4	1.3	98.8	42.5	1,088	1.4
2018	9,090	4,509	70,515	298,679	404.4	1.6	131.1	53.5	787	1.8
2019	10,217	4,124	72,728	312,280	394.1	1.3	116.1	48.8	884	1.5
Total	125,023	241,707	549,691	2,694,126	3,527.4	13.1	1,234.0	376.5	9,045	12.7

^*^Data was collected from China Health Statistics Yearbook (2012–2020).

Furthermore, the role of health literacy across cultures became more important, mainly from the perspectives of individuals, families, and communities. In 2014, National Health Literacy Promotion Action Plan (2014–2020) proposed the goal to build health promotion counties including health promotion schools, health promotion hospitals, health promotion enterprises, health promotion communities, and healthy promotion families (15). Population in these groups have common cultures, values, and needs, and share a commitment to meeting them. Cultural norms and family interpersonal relations, and values influence health literacy and help deepen the understanding and promotion of health literacy. Until 2020, health activities have covered about 40 million people every year and intend to reach 320 million people.

### Tobacco control

Tobacco use is a leading risk factor for major non-communicable diseases, and 1.4 million deaths were attributed to tobacco use in 2010 in China (19). Tobacco control is a top priority in health promotion worldwide. Serious tobacco control policies and comprehensive control actions have been actively promoted in China since the late 1970s, including adjustment of China's consumption tax on the wholesale price of cigarettes, providing a completely smoke-free environment, protecting from second-hand smoke, enforcing and revising the advertising law, carrying out smoking cessation hotline consultation, smoking cessation clinic and other services (19–21). In 2003, China signed the WHO Framework Convention on tobacco control to implement effective tobacco control policies, which provides a roadmap for effective tobacco control strategies and gives signatory countries a timetable to achieve specified milestones (22). In 2007, China issued the “Chinese Smoking Cessation Guidelines”, while in 2014 provincial government started to establish smoking cessation clinics (23, 24). In 2008, China implemented the National Tobacco Control Mass Media Campaign, including strengthening the publicity and education of tobacco and innovating the forms and content of mass-reached communications on tobacco control. The campaign advocated reinforcing people's knowledge about the health effects of smoking and second-hand tobacco smoke exposure and shaping attitudes and behaviors toward smoking bans in public places (25). In 2012, China released the “China Tobacco Control Action Plan (2012–2015)” (26). Subsequently, new national laws banned smoking in public and workplaces and cigarette advertising on TV (19). Health China Action Plan (2019–2030) proposed the goal of 80% of the population will be protected by 100% smoking bans by 2030 (17). In 2018, the smoking prevalence was 25.6% of adults aged ≥ 18, with an estimated 282 million smokers in China (27). Even though the smoking prevalence has decreased steadily in the last decades, more targeted intervention efforts should be needed to fulfill the goal of a 20% smoking rate by 2030.

### Continuous health literacy monitoring

The first nationwide survey on health literacy in China was conducted in 2008. In 2012, China has established a national continuous health literacy monitoring system. Since then, we carried out a national health literacy survey every year between July to September. Until 2021, we have already conducted 11 times national health literacy surveys. Now, the monitoring system is increasing gradually and stable which carries out health work in a scientific, standardized, and effective manner. Meanwhile, China has worked on promoting infodemic strategies and establishing a health literacy monitoring network and direct reporting system.

To better understand Chinese residents' health literacy levels and factors influencing them and prioritize intervention areas, health literacy monitoring for key groups such as the elderly and occupational groups has been included since 2022 (28). The health literacy survey was conducted using a multistage stratified sampling method. Participants of the Chinese residents' health literacy and Chinese elderly health literacy survey are aged 15–69 and 60 years and older, while participants of the Chinese key population occupational health literacy survey are workers engaged in manufacturing production and related activities. The surveys were organized and coordinated by the Health Commission, Health Education Center, and the Chinese Center for Disease Control and Prevention through household face-to-face interviews. The survey covered 1,008 communities of 336 monitoring sites in 31 provinces (excluding Hong Kong, Macao, and Taiwan).

### Fund guarantee

**Figure 5:**
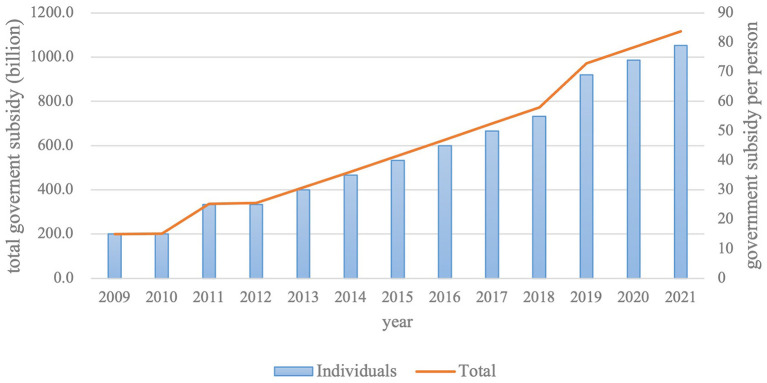
The government subsidy for National Basic Public Health Services from 2009 to 2020.

In 2012, China initiated a central subsidy program for health promotion activities. It was the first government special fund for health literacy promotion. The central subsidy was 238 million yuan in 2012, while it was 2.44 and 2.59 million yuan in 2013 and 2014, respectively. And it is used for carrying out public service advertisements, health tours, and other activities, popularizing health knowledge, and promoting the development of healthy behaviors. Additionally, China spends tens of billions of Yuan on NBPHS every year. From 2009 to 2021, the total government subsidy for NBPHS increased from 200.2 billion yuan to 1,117.2 billion yuan, with of 16.21% compound annual growth rate, while the personal subsidy increased from 15 yuan to 79 yuan, with of 15.61% compound annual growth rate (Supplementary Table 2, Figure 5).

## Discussion

This paper aimed to provide a concise overview of the characteristics and changing profile of the health literacy level from 2008 to 2020 in China. We identified the key milestones and specific practices for the promotion of health literacy advocated in China. Health literacy levels increased from 6.48 to 23.15%. Though China has made considerable progress in improving health literacy in the last decade, there was only about one-quarter of the population mastered basic health knowledge and skills in 2020. Additionally, it is positively related to the social development index. And geographic disparities were substantial, with eastern coastal provinces performing better than central and western provinces, and cities performing better than rural areas. The three aspects and six dimensions of health literacy level increased over years. However, clear disparities among them were found. Among the three aspects, the BKA literacy lever was the highest, followed by HRS, while the HBL literacy level was the lowest. Concerning the six dimensions, CD and PMC literacy was relatively low.

**Figure 6:**
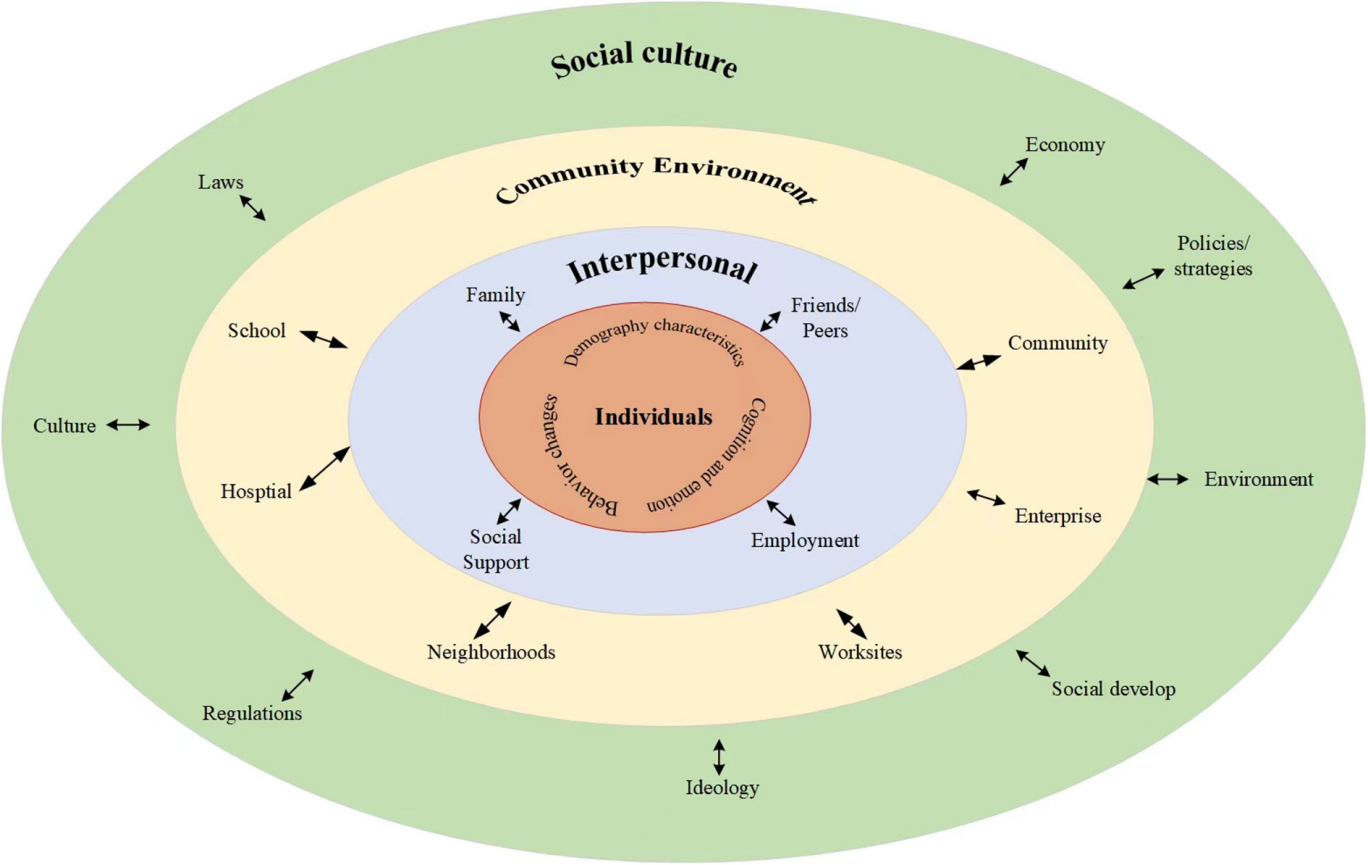
Social ecosystem theory-based framework for improving health literacy.

An effective response to health literacy issues could be more helpful for improving health outcomes and reducing health inequities. Social ecosystem theory (SET) has been increasingly used to address health issues and prevention programs (29). In this section, we proposed a SET-based framework for improving health literacy that focuses on both individual and social environment factors (Figure 6). We also presented some multi-level actionable recommendations. This framework is visualized as four concentric circles, which inform corresponding intervention strategies. From micro to macro levels, the four circles are individuals, interpersonal, community, and social culture.

The innermost circle is the individual level. It represents the individual's demography, cognition and emotion, and behavior changes. These characteristics are positively related to health literacy. Studies revealed that adults aged 25–34 had higher health literacy levels than older-aged 65–69, individuals with lower education levels and economic status, and higher risk behavior had lower health literacy levels (30–32). Currently, health literacy interventions mostly focused on 15–69 years people with higher levels of education, especially city citizens. Interventions for patients with high health needs, special groups, and their families have just started (33). All of these suggested developing more targeted and stratified literacy improvement goals and paying more attention to the age difference, geographic and education disparities, high health needs, special groups, and individuals' preferences (34, 35). Our study revealed that HBL literacy was the lowest among the three aspects, while CD and PMC literacy were relatively low among the six dimensions. And this indicated the importance of strengthening the understanding of healthy lifestyles and behaviors, chronic disease prevention, and basic medical care and treatment. Health education is always the most important and directed toward improving health literacy. Therefore, we highlighted promoting healthy lifestyles and behaviors linked to the risk of contracting the chronic disease in good ecological and social environments through strengthening the health education system and early intervention and popularizing health knowledge.

The second circle is the interpersonal level, which represents the direct person-to-person interaction, such as social support, family, friends and peers, and employment. Health decisions are often not made by individuals. They need a supportive environment that enables them to make informed health decisions and lifestyle choices. Often, families, peer groups, and employment are the primary sources of health information. These sources can be useful in developing functional health literacy skills to the selection of products and services. Additionally, families shape personality traits, and people with different personalities will adopt different lifestyles, resulting in variations in health outcomes (36). Family engagement is critical to fostering positive health outcomes (37). We recommended building effective principles of family engagement and capitalizing on their intrinsic motivation for improving the whole family's health.

The third circle is the community level, which involved formal or informal social structures, such as communities, schools, hospitals, enterprises, neighborhoods, and worksites. Communities are key settings for health literacy. People make daily health-related decisions in their homes and communities. It is essential that interventions are embedded within the environment where people live, work, and play to improve population health and reduce health disparities (38). We highly recommend community health education since the importance of concrete and efficient community action in setting priorities for health, making decisions, planning strategies, and implementing them to achieve better health. Further, popularizing health knowledge through face-to-face community health education activities and focusing on health literacy-friendliness of the various settings in which people live, play, and work are effective ways of improving health literacy (4, 31).

The outermost circle is the society level, which represents the economy, policies strategies, environment, ideology, culture, laws, and regulations. Our study showed that, in China, there were considerable differences in performance between eastern coastal provinces, central and western provinces, as well as cities and rural areas. However, these conclusions were made without considering the sociodemographic factors. Rurality may not be the only factor contributing to health literacy differences, as socioeconomic status and access to healthcare resources may also be important. Some published studies demonstrated that after controlling for confounders, rural and urban groups were not significantly different (39). Consequently, evidence-based studies are needed to investigate the true association between health literacy and geographic distribution. Evidence-based interventions should be developed, with rural populations in particular in need of improvement. Importantly, we highly advocate implementing comprehensive, whole-of-government, multisectoral policies and strategies for improving health literacy, This included promoting economic growth, increasing public investment in public goods and services, developing health-related laws and regulations, establishing state-supported health insurance schemes to increase population coverage, promoting public participation, advocating multisectoral coordination and collaboration, creating community health-supporting environments, and promoting a green lifestyle (40–43).

Additionally, the internet has already affected Chinese residents. Online information sources are increasingly used by citizens caring for their own and their Families' needs. It was estimated by the end of 2020 that 989 million Chinese people had internet access, with internet penetration has reached 70.4%. And wide internet coverage has been achieved in rural areas, and the proportion of national poverty-stricken villages with access to fiber optics increased to 98% (44). Internet literacy was positively associated with self-efficacy in utilizing eHealth. However, it is no longer the primary determinant of eHealth literacy competencies for adults who are tech-savvy users (45). We strongly recommended “Internet+” intervention on health literacy, since Internet Plus health care in China became an emerging health service model aimed at increasing access and improving the quality of health care delivery (46). Until 2020, a total of 268 internet hospitals in China have been granted official licenses (47). They primarily provided medical care to patients with common diseases or chronic diseases and to remote and rural patients who do not have access to medical care (47).

General literacy is defined as “the ability to read and write”. It is more basic and justifies your skills of reading and writing and not necessarily understanding it. Health literacy is more in-depth and is centered on the health field. Health literacy affects a person's ability to accurately search for and use health information and adopt healthier behaviors. There are nearly three-quarters of Chinese, about half of all Europeans, at least 88% of US adults, and 55.3% of Southeast Asians have poor health literacy skills (4, 7, 48, 49). Although we had made considerable efforts to promote health literacy worldwide, improving health literacy remains a critical component of achieving the Global Millennium Development Goals. A global effort to improve health literacy will be required. We strongly recommend adopting a whole-of-society approach that involves the participation of the entire ecosystem around the targeted population. First, establish a health management model and long-term working mechanism with personal responsibility, interpersonal assistance, social support, and government guidance. Second, improve health outcomes and provide access to health services for people with different health needs. Thirdly address the changing health literacy needs of individuals and communities using community wisdom, cultural systems, and local corresponding health literacy needs. Fourth, engage multiple stakeholders (individuals, government, education sector, workplaces and businesses, and community organizations) at all levels of society in the development and implementation of interventions. Moreover, pay more attention to the improvement at all levels of the health system and continuous improvement in health literacy through changes to the environment, practice, culture, and policy.

## Author contributions

YL: conceptualization, design, funding acquisition, software, draft writing, review and editing, and data analysis. XL: data collection, review, and analysis. JL: acquisition, analysis, or interpretation of data. HD: conceptualization, design, methodology, and project administration. CC: conceptualization, design, methodology, and writing—review and editing. All authors contributed to the article and approved the submitted version.

## Funding

This research was supported by the National Natural Science Foundation of China (Grant No. 81871455), Zhejiang Provincial Natural Science Foundation of China (Grant No. LY21G030013 and LY22H180001), and the Medical and Health Science and Technology Program of Hangzhou, China (Grant No. A20210432).

## Conflict of interest

The authors declare that the research was conducted in the absence of any commercial or financial relationships that could be construed as a potential conflict of interest.

## Publisher's note

All claims expressed in this article are solely those of the authors and do not necessarily represent those of their affiliated organizations, or those of the publisher, the editors and the reviewers. Any product that may be evaluated in this article, or claim that may be made by its manufacturer, is not guaranteed or endorsed by the publisher.
