# Supplementary material for: The development and progress of health literacy in China
Source: Front Public Health. 2022 Nov 7;10:1034907. doi: 10.3389/fpubh.2022.1034907 (PMC9676454; doi:10.3389/fpubh.2022.1034907)
Supplement: Supplementary file 1 [file Table_1.DOCX]

Supplementary Material

eFigure 1 The correlation between health literacy and social development index

eTable 1 Health literacy level and social development index by cities in 2019, in China

eTable 2 Government subsidy for NBPHS and the total health expenses from 2009 to 2020, in China


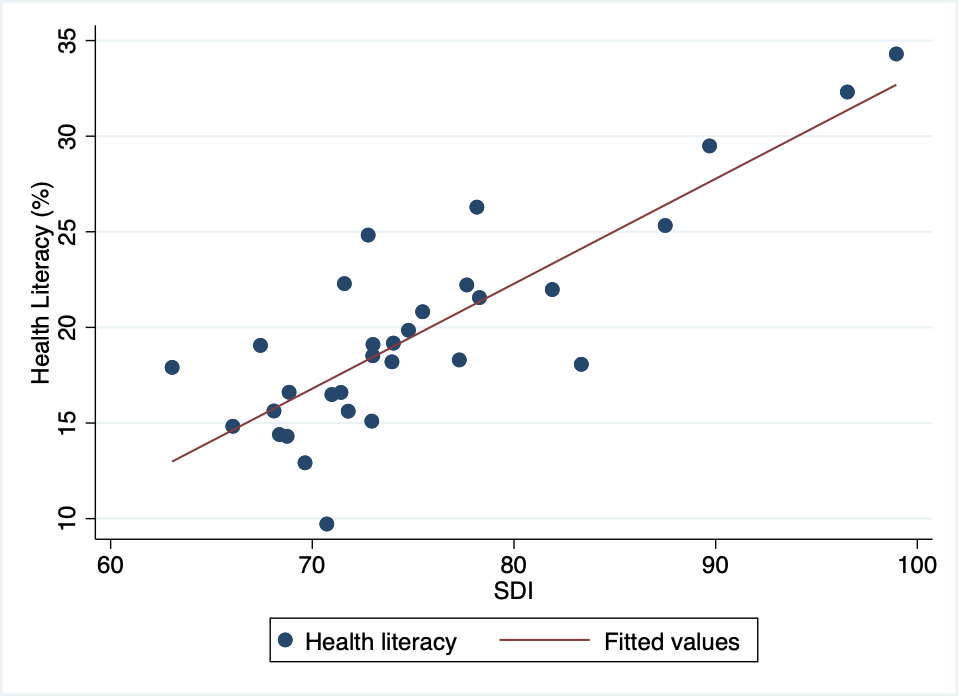


eFgiure 1 The correlation between health literacy and social development index

eTable 1 Health literacy level and social development index by cities in 2019, in China

| City | Health literacy | SDI |
| --- | --- | --- |
| Beijing | 34.30 | 98.96 |
| Shanghai | 32.31 | 96.53 |
| Zhejiang | 29.49 | 89.70 |
| Tianjin | 26.29 | 78.16 |
| Jiangsu | 25.33 | 87.50 |
| Anhui | 24.83 | 72.77 |
| Liaoning | 22.29 | 71.59 |
| Hubei | 22.22 | 77.66 |
| Fujian | 21.98 | 81.90 |
| Shandong | 21.56 | 78.29 |
| Chongqing | 20.82 | 75.47 |
| Jiangxi | 19.85 | 74.77 |
| Hebei | 19.18 | 74.02 |
| Henan | 19.11 | 73.01 |
| Heilongjiang | 19.06 | 67.43 |
| Sichuan | 18.52 | 73.00 |
| Hunan | 18.30 | 77.29 |
| Henan | 18.20 | 73.95 |
| Guangdong | 18.07 | 83.34 |
| Jilin | 17.91 | 63.05 |
| Ningxia | 16.61 | 68.85 |
| Xizang | 16.60 | 71.42 |
| Yunnan | 16.49 | 70.97 |
| Guizhou | 15.63 | 68.10 |
| Neimenggu | 15.62 | 71.78 |
| Shanxi | 15.10 | 72.95 |
| Gansu | 14.83 | 66.06 |
| Guangxi | 14.40 | 68.37 |
| Qinghai | 14.31 | 68.75 |
| Shanxi | 12.92 | 69.64 |
| Xinjiang | 9.72 | 70.72 |

eTable 2 Government subsidy for NBPHS and the total health expenses from 2009 to 2020, in China

| Year | Government subsidy for NBPHS | | Total health expenses (billion) |
| --- | --- | --- | --- |
|  | Individuals (yuan) | Total (billion) |  |
| 2009 | 15 | 200.2 | 17541.9 |
| 2010 | 15 | 201.1 | 19980.4 |
| 2011 | 25 | 337.3 | 24345.9 |
| 2012 | 25 | 339.8 | 28119.0 |
| 2013 | 30 | 410.2 | 31669.0 |
| 2014 | 35 | 481.8 | 35312.4 |
| 2015 | 40 | 553.3 | 40974.6 |
| 2016 | 45 | 626.5 | 46344.9 |
| 2017 | 50 | 700.1 | 52598.3 |
| 2018 | 55 | 773.0 | 59121.9 |
| 2019 | 69 | 973.0 | 65841.4 |
| 2020 | 74 | 1045.0 | 72175.0 |

Note: NBPHS means the “National Basic Public Health Services”.
